# The potential for Scotch Malt Whisky flavour diversification by yeast

**DOI:** 10.1093/femsyr/foae017

**Published:** 2024-04-29

**Authors:** Martina Daute, Frances Jack, Graeme Walker

**Affiliations:** Division of Engineering and Food Sciences, School of Applied Sciences, Abertay University, Bell St, DD1 1HG, Dundee, Scotland; The Scotch Whisky Research Institute, Research Ave N, EH14 4AP, Edinburgh, Scotland; The Scotch Whisky Research Institute, Research Ave N, EH14 4AP, Edinburgh, Scotland; Division of Engineering and Food Sciences, School of Applied Sciences, Abertay University, Bell St, DD1 1HG, Dundee, Scotland

**Keywords:** yeast, fermentation, whisky, Scotch Whisky, non-conventional yeast, non-*Saccharomyces*, distilled spirits

## Abstract

Scotch Whisky, a product of high importance to Scotland, has gained global approval for its distinctive qualities derived from the traditional production process, which is defined in law. However, ongoing research continuously enhances Scotch Whisky production and is fostering a diversification of flavour profiles. To be classified as Scotch Whisky, the final spirit needs to retain the aroma and taste of ‘Scotch’. While each production step contributes significantly to whisky flavour—from malt preparation and mashing to fermentation, distillation, and maturation—the impact of yeast during fermentation is crucially important. Not only does the yeast convert the sugar to alcohol, it also produces important volatile compounds, e.g. esters and higher alcohols, that contribute to the final flavour profile of whisky. The yeast chosen for whisky fermentations can significantly influence whisky flavour, so the yeast strain employed is of high importance. This review explores the role of yeast in Scotch Whisky production and its influence on flavour diversification. Furthermore, an extensive examination of nonconventional yeasts employed in brewing and winemaking is undertaken to assess their potential suitability for adoption as Scotch Whisky yeast strains, followed by a review of methods for evaluating new yeast strains.

## Teaser

Research into Scotch Whisky production highlights the critical role of unconventional yeast for its production, important factor for the diversification of its distinct taste, and global appeal.

## Introduction

In Scotland, the production of whisky is important for the revenue of the country as well in attracting visitors. There are 148 operational Scotch Whisky distilleries with a contribution of £7.1 billion to the UK’s economy in 2020. This results in Scotch Whisky being responsible for 77% of Scottish food and beverage exports. Many of these distilleries have visitor centres, attracting over 2.2 million visitors per year (The Scotch Whisky Association 2023) supporting Scotland’s economy and tourism. The size of a malt whisky distillery is variable, with Glenlivet and Glenfiddich having the largest production capacity of 21 000 000 LPA (litres of pure alcohol per annum) and Dornoch one of the smallest with 25 000 LPA (Gordon 2022).

It is not only the revenue, i.e. important for Scotland, but the country is also proud of this quality product and its long history as evidenced by its protection under the Scotch Whisky Regulation (2009). Nevertheless, there is a steady stream of innovation and research, with on average more than 12 000 new publications every year.

Following the trend of investigating the influence of nonconventional or non-*Saccharomyces* yeast in wine (e.g. Jolly et al. 2006, Roudil et al. 2019) and beer (e.g. Basso et al. 2016, Bellut and Arendt 2019, Larroque et al. 2021), recent research has also been initiated for Scotch Whisky (Daute 2021). The flavour of Scotch whisky emanates from several sources during the production from raw materials (grains and water), mashing, fermentation, distillation (design and conditions), and maturation (time and cask). However, the choice of yeast strain is one of the most important factors affecting the organoleptic properties of new make spirit and young whiskies. This is primarily due to the production of high levels of volatile congeners including esters and higher alcohols. In more matured whiskies, the maturation conditions, including choice of oak cask and the duration of ageing, act to provide desirable flavours and reduce undesirable off-flavours in the spirit (Wanikawa 2020). We propose that unconventional yeasts can be exploited as novel drivers for distilled spirit flavour differentiation. This paper reviews the use of yeast in Scotch Whisky fermentations, the effect of yeast on spirit flavour, and the potential of non-*Saccharomyces* yeast for production in the future. While whisky is produced worldwide, this review focuses primarily on Scotch Malt Whisky.

## An overview of Scotch Malt Whisky production

**Figure 1.**
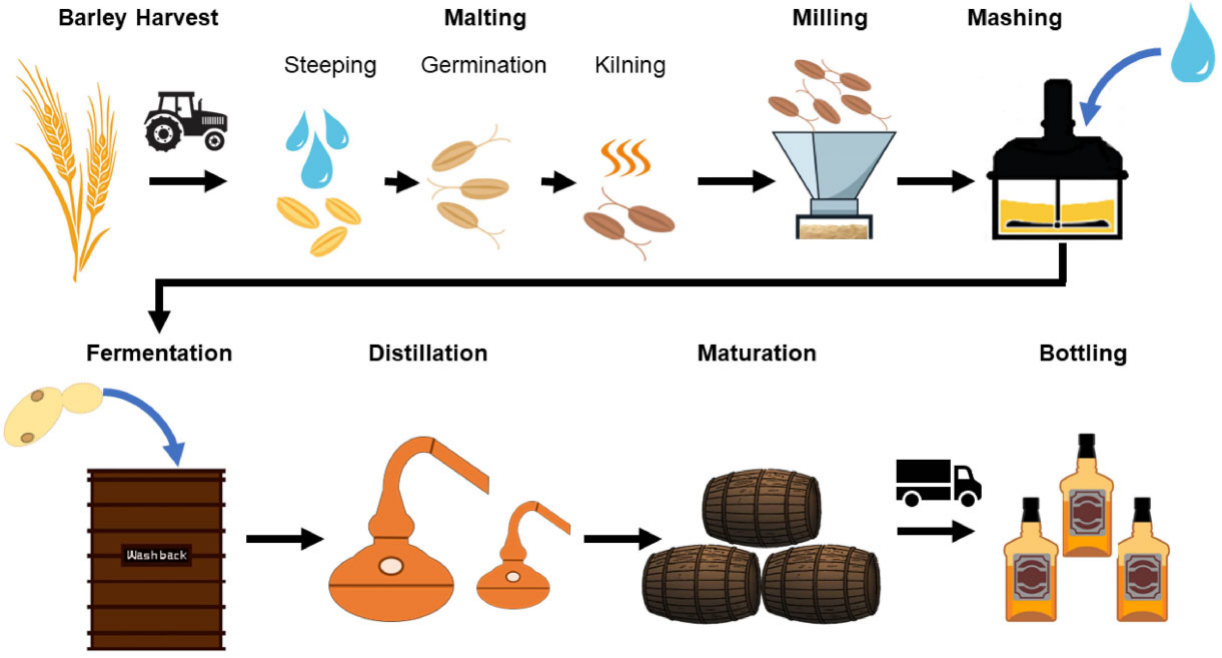
Illustrating Scotch Malt Whisky production.

Scotch Malt Whisky production is strictly regulated by The Scotch Whisky Regulations (2009). It must be produced and matured in Scotland from only three ingredients: water, malted barley, and yeast, with plain caramel colouring allowed in some cases. When making any modifications to the production methods, it is vital to ensure that the resulting spirit has the typical aroma and taste of Scotch (The Scotch Whisky Regulations 2009). The production process is summarized in Fig. 1.

Malt whisky production starts with the malting of barley to break down starch and proteins into fermentable sugars and amino acids. This occurs by letting the barley germinate and then drying (kilning) it to guarantee a stable product (Bathgate 2016, Mosher and Trantham 2017). The final malt specifications are important for production efficiency, processability, spirit quality, flavour, and yeast performance (Bringhurst and Brosnan 2014, Bringhurst 2015, Marčiulionytė et al. 2022). The malt is mashed with hot water to further break down starch via malt-derived enzymes. Use of extraneous amylolytic enzymes is not permitted (The Scotch Whisky Regulations 2009).

The resulting liquid (wort) is cooled (20–25°C) and transferred into either wooden or stainless steel washbacks (fermenters), where yeast is added to start the fermentation with a common pitching rate of 2–4 × 10^7^ cells/ml (Watson 1981, Bringhurst and Brosnan 2014, Russell and Stewart 2014, Walker and Hill 2016). Commonly, the wort for Scotch Whisky has an original gravity of 1060–1080° (Russell and Stewart 2014). In contrast to brewing, the wort is not boiled, allowing the further hydrolysis of starch and in a later stage the growth of other microorganisms. During the fermentation, yeast converts malt-derived sugars (primarily maltose) into carbon dioxide, ethanol, and flavour compounds (congeners) that will transpire into the final distilled product. The fermentation temperature rises naturally to 33°C through the metabolic activity of the yeast (Watson 1981, Walker and Hill 2016). After 30 h, the fermentation is largely complete and this can be detected by monitoring a decrease in the specific gravity of the wash (fermented wort) to 975°, resulting in a liquid with an alcohol by volume (ABV) of 8%–10% v/v and a drop in pH to 4.2. Most malt whisky distilleries extend the fermentation time to allow microorganisms (mainly lactic acid bacteria) to produce more congeners (Russell and Stewart 2014, Walker and Hill 2016).

Next, the ethanol and congeners are concentrated by a double distillation in traditional copper pot stills. The first distillation stops when the resulting distillate’s alcohol content is below 1% v/v ABV, leading to an ABV of 20%–25% v/v. This distillate fraction is referred to as ‘low wines’ (Nicol 2014, Piggott 2017). The second distillation is split into three sections: foreshots/head, spirit cut/heart, and feints/tails based on the ABV and congener concentration. The feints and head cut will be recirculated and included in the next distillation. Only the spirit cut with an ABV of around 70% v/v is used for the maturation which must last for at least 3 years in oak casks (The Scotch Whisky Regulations 2009). Some distilleries use a triple still set-up to produce their whiskies or for special releases, which was more common in the past due to lower alcohol yields during fermentation (Glen 1969, Wanikawa 2020). Triple-distillation is commonly conducted for production of Irish whiskeys, but an example of a distillery in Scotland where it is practised is Auchentoshan (Auchentoshan 2019). The previous cask use (Piggott et al. 1993, Mosedale 1995), as well as cask and storage conditions (Clyne et al. 1993, Spillman et al. 2004, Roullier-Gall et al. 2020) influence the final flavour. The flavour profile evolves from pungent, oily, sulphury, and sour to more mellow, vanilla, and sweet notes which constitute the main flavour characteristics of Scotch Malt Whisky.

## History of yeast use in Scotch Whisky

Reusing yeast in Scotch Whisky fermentation is not practised because the wort is not boiled or sterilized in any other way, which increases the risk of microbial contamination (Dolan 1976, Walker et al. 2011a, Russell and Stewart 2014, Walker and Hill 2016). Additionally, leaving the yeast in the wash during distillation contributes to the distinct flavour characteristics of the resultant spirit (Suomalainen and Lehtonen 1979). Today, Scotch Whisky distillers usually do not propagate their yeast, buying them instead from yeast supply companies (Walker et al. 2011b, Walker and Hill 2016). With very few exceptions, most strains used in the distilling industry in Scotland are *Saccharomyces cerevisiae*.

Historically, spent brewing yeast was used due to its affordability and convenience (Russell and Stewart 2014). Records suggest that as early as 1833, Scotch Whisky distillers produced separate yeast to increase the yield. In 1920, the Distillers Company Limited introduced the first commercially available pure standard yeast for Scotch Whisky (Frey 1930). This did not stop distilleries from sourcing their yeast from local breweries or producing it themselves until the 1950’s. With the introduction of M strain or M-type (interspecies hybrid between *S. cerevisiae* and *S. cerevisiae* var. *diastaticus*) by DCL Yeast Ltd (now Kerry Biosciences) in 1952, this changed, and it became the standard distilling yeast (Watson 1981). At this time, yeast was used in combination with 30%–50% w/w recycled brewer’s yeast. This resulted in increased alcohol yield, overall fermentation performance, and greater flavour complexity (Dolan 1976, Noguchi et al. 2008, Yomo et al. 2008, Walker et al. 2011a,b, Walker and Hill 2016). This situation changed again in the late 1990’s/mid 2000 due to the closure of many of the larger breweries in Scotland and subsequent reduced availability of brewer’s yeast. As a result, most distilleries switched to relying mainly on using commercially available Scotch Whisky yeast (Walker et al. 2011a, Stewart et al. 2013, Walker and Hill 2016, Bathgate 2019).

While the M-type yeast has changed over the years, it is still declared as one of the standards in the Scotch Whisky industry together with MX (Kerry Bio-Science), Pinnacle (Mauri/AB Biotek), and DistillaMax (Lallemand Inc.). All of these strains belonging to the species of *S. cerevisiae* (Watson 1981, Walker et al. 2011a,b, Walker and Hill 2016). These contemporary distilling yeasts are well-adapted to fermenting cereal-based wort, being able to convert larger starch-derived sugars and dextrin more efficiently into ethanol and additionally being better able to withstand different physical and chemical environmental stresses (Russell and Stewart 2014). Yeast from supply companies is provided in different formats for distilling such as dried, creamed, caked, or stabilized liquid. Each distillery selects the format based on their capability for transport, storage, and fermentation capacity (Watson 1981, Russell and Stewart 2014, Walker and Hill 2016).

## Variety of yeast species and their application in alcoholic beverages

All alcoholic beverages, distilled or not, have one thing in common: yeast. The most commonly used yeast species *S. cerevisiae* has been used by humans for centuries (McGovern et al. 1996, 2004). The fermentation of food products was discovered accidentally by grapes starting to spontaneously ferment due to naturally occurring yeast. Microorganisms, including yeasts, were discovered in 1680 by Antoine van Leeuwenhoek followed by further studies of fermentation in 1789 by Antoine Lavoisier (Mortimer 2000, Chambers and Pretorius 2010).

Yeasts belong to the kingdom of fungi and are present in the divisions of ascomycetous, basidiomycetous, and deuteromycetous fungi. Often, only the subphylum of Saccharomycotina is considered as ‘real’ yeast. Overall, yeast are eukaryotic, unicellular organisms that got their name based on their ability to ferment with a meaning of ‘foam’ and ‘to rise’ (Kurtzman et al. 2011a). For the industrial use of yeast, they are often separated into *Saccharomyces* spp., yeast that have been used for many years for brewing or baking and ‘non-conventional’ yeast or non-*Saccharomyces* yeast, which came into the focus of industry only relatively recently. These yeasts were frequently branded as spoilage wild yeasts (Legan and Voysey 1991, Fleet 2011, Blomqvist and Passoth 2015, Shimotsu et al. 2015) and it was assumed that they were less effective in their fermentation performance than to *S. cerevisiae*. Table 1 summarizes the strengths and weaknesses of *S. cerevisiae* and non-*Saccharomyces* yeasts in distilled spirits production.

**Table 1.** Comparison of *S. cerevisiae* and non-*Saccharomyces* yeasts for distilled spirits production.

*S. cerevisiae*		non-*Saccharomyces*	
Strengths	Weaknesses	Strengths	Weaknesses
Ferment sugars^1^	Only metabolize mono-, di-, and tri-hexoses (no starch or lactose)^8^	Wide variety	Mostly Crabtree-negative^13^
High stress tolerance^1^	Limited genetic variability	Different sugar metabolism^9^	Some yeasts are opportunistically pathogenic
Wide temperature tolerance^1^	Not regarded as thermophilic^2^	Selected yeasts have high alcohol production^10^	Only selected yeasts recognized as generally regarded as safe (GRAS)
High alcohol tolerance (∼14%–15% v/v)^2^	Room for improvement with industrial strains	Different metabolic pathways^11^	Limited research
High sugar tolerance^3^	Weak osmotolerance in some strains^2^	Diversification of congener production^11^	Produce low/no alcohol^9^
Crabtree-positive/fermenting in the presence of high sugar levels and oxygen^4^	Crabtree effect needs. To be avoided for optimal yeast propagation	Provide new congeners such as: 4-ethylguaiacol^12^	Incomplete fermentation^9^
Generally regarded as safe (GRAS)^5^			
Well-researched^6^			
Widely used^7^			
Metabolic pathways known^7^			
Easy to culture			

Superscripted numbers in the table represent following references:

1 Torija et al. (2003) and Parviz et al. (2011).

2 Ghareib et al. (1988), Hosaka et al. (1998), Pina et al. (2004), Osho (2005), Walker and Hill (2016), and Morard et al. (2019).

3 Osho (2005), Pereira et al. (2011), and Tao et al. (2012).

4 De Deken (1966), Alexander and Jeffries (1990), Quirós et al. (2014), and Perez-Samper et al. (2018).

5 Walker and Hill (2016).

6 Botstein et al. (1997), Legras et al. (2007), Liti (2015), Bilinski et al. (2017), and Alexander (2018).

7 Walker (2009), Wang et al. (2012), Goddard and Greig (2015), Ramazzotti et al. (2019), and Meriggi et al. (2020).

8 Pretorius et al. (2003), Domingues et al. (2010), and Walker and Hill (2016).

9 Petit et al. (2000), Knoshaug et al. (2009), Rodicio and Heinisch (2009), Basso et al. (2016), Varela (2016), Bellut and Arendt (2019), and Mehlomakulu et al. (2021).

10 Pina et al. (2004).

11 Romano et al. (1992), Lambrechts and Pretorius (2000), Zohre and Erten (2002), Clemente-Jimenez et al. (2004), Domizio et al. (2011), and Magyar et al. (2014).

12 Heresztyn (1986), Shinohara et al. (2000), and Coghe et al. (2004).

13 De Deken (1966), Alexander and Jeffries (1990), Bellaver et al. (2004), Gonzalez et al. (2013), and Contreras et al. (2015).

Recent research has shown that non-*Saccharomyces* yeasts have more potential than previously anticipated in utilizing different substrates. These include *Kluyveromyces marxianus* converting cheese whey into vodka and bioethanol (Grba et al. 2002, Fonseca et al. 2008) or *Saccharomycodes ludwigii* and *Pichia kluyveri* to produce low-alcohol beer (Myncke et al. 2023) or *Torulaspora* and *Metchnikowia* spp. producing different flavour profiles in wine or beer (Bellut and Arendt 2019, Roudil et al. 2019).

## Experimental data of Scotch Whisky fermentation

Exploration of novel distilling yeasts for the Scotch Whisky industry is not a new task, with early initiatives, such as by Chivas Brothers in 1981, involving the establishment of a yeast production plant to produce alternative and secondary yeast strains (Watson 1981). The analytical focus at that time extended to assessing the influence of different fermentation parameters, including temperature, suspended solids, alcohol tolerance, and bacterial contamination (Merritt 1966, 1967, Dolan 1976, Ramsay and Berry 1983, 1984, Okolo et al. 1990, Daute et al. 2021a). The primary emphasis remained on the development of high ethanol-yielding yeasts, with distillers relying on the distillation process to ensure the production of an acceptable spirit (Dolan 1976, Watson 1981, Berbert de Amorim Neto et al. 2009), or comparing different commercial yeast products, formats, and pitching rates (Reid et al. 2023, Spasova et al. 2023, Waymark and Hill 2021).

Notably, limited attention has been given over the years to investigating the influence of yeasts on the flavour profile of Scotch Whisky. Previous research predominantly explored distinctions among commercial *S. cerevisiae* yeasts (Ensor et al. 2015, Miles 2015, Ekins et al. 2018). Some non-distilling yeasts used in co-cultures with distilling strains demonstrated a reduction in yield but an increase in estery (fruity) flavours (Miles 2015). Co-fermentation with pure cultures of brewing yeast exhibited flavour enhancement (Wanikawa et al. 2004, Noguchi et al. 2008, Yomo et al. 2008), while the use of bioethanol strains resulted in spirits with flavours comparable to whisky distilling yeast (Berbert de Amorim Neto et al. 2008, 2009, Daute 2021).

To date, very few commercial Scotch Whiskies have prominently featured the use of nonconventional yeasts in their marketing. *Schizosaccharomyces pombe*: Glen Elgin 1998—18-year-old Special Release 2017 (Master of Malt 2021) and the Glenmorangie Allta, produced with a local wild yeast from Cadboll barley named *Sacchaormyces diaemath* (Broom 2019). Nevertheless, some craft-distillers investigate and isolate wild yeasts from the area around the distillery or their raw materials to create new products with alternative flavours, as observed at Lindores Abbey Distillery (Burke et al. 2014, 2015, Walker and Hill 2016).

As Scotch Whisky fermentations are not sterile processes, microorganisms other than the pitched distilling yeast strain influence the fermentation flavour of the new make spirit (Watson 1981, Walker and Hill 2016). A distilling yeast with a poor sugar-to-alcohol conversion results in more residual sugars, giving other microorganisms a higher chance to grow and potentially have a deleterious influence on product quality. These microorganisms enter the process through raw materials, the environment (air and dust), or production equipment: water used in different production steps can bring in low levels of wild *Bacillus* spp., and Enterobacteria (Guild et al. 1985, Wilson 2014). Barley is a source of a wide variety of bacteria and wild yeast including *Candida* spp., *Cryptococcus* spp., *Hansenula* spp., *Rhodotorula* spp., and *Saccharomyces* spp. (Flannigan 1999, Noots et al. 1999, Van Nierop et al. 2006, Justé et al. 2011). During malting the variety of bacteria decreases with a dominance of lactic acid bacteria. Nevertheless, a wide variety of wild yeast is still present, consisting among others, of *Aureobasidium* spp., *Candida* spp., *Cryptococcus* spp, *Debaryomyces* spp., *Issatchenkia* spp.*, Kluyveromyces* spp., *Pichia* spp., *Rhodotorula* spp. (Flannigan 1999, O’Sullivan et al. 1999, Booysen et al. 2002, Laitila et al. 2006, 2011, Justé et al. 2011). During mashing, the overall wild yeast count is drastically reduced. As for bacteria, the microflora consists mostly of lactic acid bacteria, acetic acid bacteria, and *Gluconobacter* spp. (Guild et al. 1985, O’Sullivan et al. 1999, Wilson 2014). In the subsequent production step, fermentation, the added yeast will be the dominant microorganism. Only low levels of other wild yeast will still be present, lactic acid bacteria and rarely acetic acid bacteria, *Zymomonas* spp., and *Pediococcus* spp. Often the concentration of these increase with extended fermentation time (Makanjuola and Springham 1984, Priest and Barker 2010, Wilson 2014).

## Yeast strain improvement

The primary objectives for distilling yeast strains encompass achieving a high sugar-to-alcohol conversion (exceeding 90%), minimizing the production of off-flavours, exhibiting high-stress tolerance, ensuring high viability, and demonstrating efficient rehydration efficiency (Pretorius et al. 2003, Walker et al. 2011a). In addition to this, further development of new Scotch Whisky distilling strains is focused on the following desired attributes:

high tolerance to ethanol, heat, low pH, osmotic pressure, and high sugar concentration
rapid fermentation of the wort sugars glucose, maltose, and maltotriose
production of appropriate congeners
high flavour consistency
high viability/vitality
a short lag phase
minimal yeast biomass requirement
competitiveness with other microorganisms
high endurance under various transport conditions
culture stability
non-flocculent
Generally Recognized as Safe (GRAS) or Qualified Presumption of Safety (QPS) status

*Adapted from* Walker et al. (2011a,b), Russell and Stewart (2014), and Walker and Hill (2016).

Four approaches are commonly employed to attain these goals in new distilling strains: natural biodiversity, selection through methods such as mutagenesis (Liu et al. 2008, 2018b) and hybridization/breeding (Bellon et al. 2013, Gibson et al. 2017, Gallone et al. 2019, Stewart 2019), adaptive evolution (Saerens et al. 2010, Gallone et al. 2016, 2018, Barbosa et al. 2018, Gibson et al. 2020), and genetic modification (GM)/gene editing. The current stance of the Scottish Government and public opinion opposes the use of GM crops, leading to the exclusion of these or other GMOs (genetically modified organisms) in food production (Stewart et al. 2013, Scottish Government 2020, Science and Advice for Scottish Agriculture 2021). Consequently, GM and asexual hybridization methods like protoplast fusion, often considered as GM (Husby 2007) are currently not employed by the Scotch Whisky industry for yeast strain improvement.

A common approach in industry is to either start with an already commercially available yeast strain, screen a strain collection, or collect wild samples to exploit the natural biodiversity. For example, a wide variety of *Saccharomyces* spp. and non-*Saccharomyces* yeasts can be isolated from different habitats (Alsammar and Delneri 2020, Hutzler et al. 2021, Pinto et al. 2022, Piraine et al. 2022, Iturritxa et al. 2023), with several *S. cerevisiae* isolations often associated with human habitats (Fay and Benavides 2005). Different selection techniques and media have been used for the isolation of specific yeasts. The next step involves further modification and adaptation of the selected yeast strain. For this, a combination of breeding, mutagenesis, and adaptive evolution or a combination thereof can be used. Yeast breeding can integrate traits from different strains and, potentially, closely related species, and this requires further work to stabilize the traits in the final yeast strain (Krogerus et al. 2017). Mutagenesis involves exposing the yeast to mutagenic materials or UV-rays to elevate the mutation rate, and resultant yeasts are screened for specific phenotypes. Yeasts exhibiting desired traits are selected for subsequent rounds until the yeast possesses improved characteristics, which can be again bred with a different strain. A similar principle is used for directed evolution, the yeast is placed in an environment that applies an evolutionary pressure, such as steady increase of sugar concentrations to guide the direction of mutation, enhancing the yeast’s survival in an artificially adjusted environment, and thereby improving physiological traits like sugar metabolism or flavour development (Dequin 2001, Liu et al. 2008). Recently, the Carlsberg Research Laboratory has introduced a new technique called FIND-IT to accelerate the identification of yeast and other organisms with desired mutations, allowing to screen for single nucleotide polymorphisms (SNP) (Knudsen et al. 2022).

Additional promising avenues for further research in whisky fermentations include exploring amylolytic yeasts for more efficient starch breakdown (Laluce et al. 1988, Pretorius et al. 2003, Cheng et al. 2011, Walker et al. 2011a) or further elaborating flavour profiles, e.g. using POF+ (phenolic off-flavour positive) yeasts to impart phenolic and spicy notes (Heresztyn 1986, Coghe et al. 2004). Further research into non-*Saccharomyces* yeasts for industrial fermentations is expected. Recent findings comparing the flavour profile of wash, low wines, and new make spirit of different yeast strains showed that the key flavour notes are stable throughout these production steps. This finding will support the development of new yeast strains by reducing the time needed for sample preparation by eliminating the need for a double distillation for early yeast screening rounds (Daute et al. 2023). Together with the finding that congener profiling of wort by gas chromatography–mass spectrometry (GC–MS) gives comparable data to the sensory evaluation, this could further reduce the time by not requiring a sensorial evaluation of samples in early screening steps (Daute et al. 2021b).

## Non-conventional yeast used for distilled spirits

In the production of neutral spirits such as vodka, gin, or bioethanol, yeast selection is not a primary consideration because the final product undergoes extensive purification, and most yeast derived congeners are undesired in the final product. Consequently, efficiency becomes the primary factor, leading to the preference for highly adapted *S. cerevisiae* strains with robust stress tolerance (Pauley and Maskell 2017, Black and Walker 2023, Spasova et al. 2023) instead of nonconventional flavourful yeast.

In contrast to Scotch Whisky production, the use of a variety of yeast strains is more commonplace in other distilled spirit industries. For example, Bourbon and Tennessee whiskey distilleries often cultivate their own proprietary yeast strains (Smith 2017). Historically, after the increased availability of commercial yeast, Scotch Whisky producers hesitated to adopt this practice, deeming it economically impractical due to concerns about quality, cost, and sustainability (Walker and Hill 2016).

The transition towards deliberately inoculated fermentations with *S. cerevisiae* marked a departure from the diversity and complexity of flavours typically associated with spontaneous fermentations (Gschaedler 2017). While wild fermentation offers potentially more complex flavours, it concurrently extends fermentation time, potentially resulting in a 40%–60% v/v decrease in alcohol yield, and higher levels of residual sugars. Despite this, some distilleries prioritize flavour over yield (Fahrasmane and Ganou-Parfait 1998, Nuñez-Guerrero et al. 2016, Portugal et al. 2017). Table 2 provides an overview of yeasts used in various distilled spirits production.

**Table 2.** Yeasts involved in the production of distilled spirits.

Product	Spontaneous fermentation	Researched non-*Saccharomyces* yeasts for flavour production
Rum	*Candida krusei, Candida stellate, Pichia membranifaciens, Saccharomyces* spp., *Schizosaccharomyces* spp., *Wickerhamomyces anomalus*^1^	
Mezcal, Tequila, fermentation of agave juice	*Candida* spp., *Dekkera bruxellensis, Hanseniaspora guilliermondii, Hanseniaspora vinae, Klockera apiculta, Kluyveromyces marxianus, Pichia kluyveri, Pichia membranifaciens, Rhodotorula* spp., *Saccharomyces cerevisiae, Torulaspora delbrueckii*^2^	*Candida krusei, Candida magnolia, Klockera africana, Klockera apiculate, Kluyveromyces marxianus, Pichia caribbica, Pichia kluyveri, Torulaspora delbrueckii, Wickerhamomyces anomalus* ^3^
Cachaça	*Candida maltose, Candida sake, Debaryomyces hansenii, Hanseniaspora uvarum, K. marxianu*s, *Pichia heimii, Pichia methanolica, Pichia subpelliculosa, Rhodotorula glutinis, Saccharomyces cerevisiae*, Schizosaccharomyces *pombe, Torulaspora delbrueckii, Wickerhamomyces anomalus*^4^	*Candida famata, Candida guillermondii, Hanseniaspora guillermondii, Hanseniaspora occidentalis, Meyerozyma caribbica, Meyerozyma guillermondii, Pichia caribbica, Pichia fermentans, Pichia subpelicullosa, Schizosaccharomyces pombe, Wickerhamomyces anomalus* ^5^
Honey-based distillates	*Lachancea fermentati, Pichia kudriavzevii, Saccharomyces cerevisiae, Wickerhamomyces anomalus, Zygosaccharomyces bailiiand, Zygosaccharomyces rouxii* ^6^	
Grape-based distillates	*Candida lactis-condensi, Hanseniaspora osmophila, Pichia galeiformis, Torulaspora delbrueckii* ^7^	
Vodka–cheese whey^8^		*Kluyveromyces marxianus*
Fruit spirit^9^		*Aureobasidium* sp., *Kluyveromyces apiculate, Lachancea thermotolerans, Torulaspora delbrueckii*

Superscripted numbers in the table represent following references:

1 Parfait and Sabin (1975), Fahrasmane et al. (1988), Lachance (1995), Fahrasmane and Ganou-Parfait (1998), and Fleet and Green (2010).

2 Lachance (1995), Arellano et al. (2008), Escalante-Minakata et al. (2008), Lappe-Oliveras et al. (2008), Soto-García et al. (2009), Verdugo Valdez et al. (2011), Páez-Lerma et al. (2013), Nolasco-Cancino et al. (2018), and Walker et al. (2019).

3 Fiore et al. (2005), Arrizon et al. (2006), Arellano et al. (2008), López-Alvarez et al. (2012), Segura-García et al. (2015), and Nuñez-Guerrero et al. (2016).

4 Morais et al. (1997), Pataro et al. (2000), Schwan et al. (2001), Badotti et al. (2010), and Brexó et al. (2020).

5 Oliveira et al. (2004), Duarte et al. (2012), Amorim et al. (2016), and Portugal et al. (2017).

6 Gaglio et al. (2017).

7 Úbeda et al. (2014).

8 Walker and O’Neill (1990), Zafar and Owais (2006), Fonseca et al. (2008), Mazaheri Assadi et al. (2008), and Delshadi (2019).

9 Satora and Tuszyński (2010) and Fejzullahu et al. (2021).

Pure cultures of non-*Saccharomyces* yeasts exhibit distinct flavour profiles, often characterized by higher levels of esters or higher alcohols compared to *S. cerevisiae*. However, their fermentation performance is often poorer by comparison (Dato et al. 2005, Oliveira et al. 2005, Arellano et al. 2008, López-Alvarez et al. 2012, Segura-García et al. 2015). Therefore, a combination of a non-*Saccharomyces* strain with a commercial distilling yeast often results in increased yield and enhanced ester notes (Duarte et al. 2012, Nuñez-Guerrero et al. 2016). Optimizing non-*Saccharomyces* yeast could enhance their fermentation performance, increase ABV, and introduce unique flavours (Dato et al. 2005, Oliveira et al. 2005, Arellano et al. 2008, López-Alvarez et al. 2012, Segura-García et al. 2015). Commercial yeast strains, belonging to *S. cerevisiae*, have undergone years of optimization, and new yeast strains with improved fermentation properties, such as MG + from AB Mauri, have recently been introduced to the market (Storr and Walker 2018).

Recently, Kveik yeast, traditional Norwegian farmhouse yeast, has gained attention in brewing due to its phenolic off-flavour negativity, high fermentation rate, tolerance to high temperatures (>28°C), and classification within the *S. cerevisiae* clade (Preiss et al. 2018). This interest has extended to the distilling industry, where Kveik yeast demonstrates a fermentation pattern similar to commercial distilling yeast and a distinct flavour profile, offering the opportunity for development of new products (Dippel et al. 2022, Horstmann et al. 2023).

## Non-conventional yeast used for winemaking and brewing

Non-conventional yeasts are increasingly used in the production of nonalcoholic or low-alcoholic beverages, particularly for wine and beer. Although these yeasts produce less ethanol, they contribute different and often increased levels of congeners, resulting in an altered flavour profile of these beverages (Bellut and Arendt 2019).

In wine and beer production, selecting starter cultures is a common practice to improve control over fermentation performance, flavour, and the creation of specific products (Carrasco et al. 2001, Fernández-Espinar et al. 2001, Romano et al. 2003, Ribéreau-Gayon et al. 2006, Torrens et al. 2008, Chambers and Pretorius 2010, Schuller 2010, Garofalo et al. 2016, Capozzi et al. 2017, Berbegal et al. 2018, Vilela 2021). In the wine industry, *S. cerevisiae* strains are the predominant commercial yeast starters, resulting in most research focused on *S. cerevisiae* (Cadière et al. 2012, Tian et al. 2020) and related species such as *S. bayanus* and *S. uvarum* (Carrasco et al. 2001, Fernández-Espinar et al. 2001, Masneuf-Pomarède et al. 2010, Almeida et al. 2014, Alonso-del-Real et al. 2017). In brewing, *S. cerevisiae* strains dominate ale production, while *S. pastorianus* (a hybrid of *S. cerevisiae* and *S. eubayanus*) is prominent in lager production. Commercially offered strains also include *S. cerevisiae* and *S. uvarum* (Stewart et al. 2013, Gibson et al. 2017).

While commercial starter cultures provide consistent fermentations and flavour profiles, nonconventional yeasts offer the opportunity to diversify flavour in fermented beverages (Roudil et al. 2019, Molinet and Cubillos 2020). The introduction of commercial non-*Saccharomyces* yeasts in winemaking began in 2004 by Christian Hansen, resulting in the release of a pure *Torulaspora delbrueckii* strain in 2009 (Roudil et al. 2019, Peyer 2020). Non-*Saccharomyces* yeasts are often used in cocultures or sequential fermentations together with *Saccharomyces* yeasts to optimize sugar utilization, ethanol production, and wine flavour elaboration. Table 3 provides a list of nonconventional and non-*Saccharomyces* yeasts used in both spontaneous and controlled winemaking and brewing.

**Table 3.** List of non-conventional and non-*Saccharomyces* yeasts used in spontaneous and controlled winemaking and brewing.

	Winemaking	Brewing
**Commercial yeasts**	*Candida zemplinina, Kluyveromyces wickerhamii, Lachancea thermotolerans, Metschnikowia pulcherrima, Metschnikowia fructicola, Pichia kluyveri, Saccharomyces cerevisiae, Saccharomyces bayanus, Schizosaccharomyces pombe, Torulaspora delbrueckii, Wickerhamomyces anomalus* ^1^	*Brettanomyces* spp. [*Brettanomyces claussenii* (reclassified as *Dekkera anomala), Brettanomyces bruxellensis, Brettanomyces lambicus* (reclassified as *Dekkera bruxellensis*)], *Lachancea* spp., *Pichia kluyveri, Saccharomyces cerevisiae, Saccharomyces pastorianus, Saccharomyces uvarum* (reclassified as *Saccharomyces bayanus*)^2^
**Spontaneous fermentation**	Dominated by *Saccharomyces cerevisiae/Saccharomyces* spp., *Aureobasidium pullulans, Candida stellate, Candida zemplinina, Hanseniaspora uvarum, Issatchenkia occidentalis, Issatchenkia terricola, Kloeckera apiculate, Lachancea thermotolerans, Metschnikowia fructicola, Metschnikowia pulcherrima, Pichia fermentans, Pichia membranifaciens, Pichia kudruavzevii Rhodotorula glutinis*^3^	*Brettanomyces* spp., *Candida* spp., *Debaryomyces* spp., *Hanseniaspora uvarum, Pichia* spp., *S. dairensis, S. cerevisiae, S. bayanus, S. pastorianus, S. uvarum*^4^
**Researched nonconventional yeast**	*Brettanomyces* spp., *Candida* spp., *Hanseniaspora* spp., *Kloeckera* spp., *Metschnikowia* spp., *Pichia* spp., *Schizosaccharomyces* spp., *Starmella* spp., *Saccharomycodes* spp., *Torulaspora* spp., *Williopsis* spp., *Zygosaccharomyces* spp.^5^	Kveik yeast, *Brettanomyces anomalus, Dekkera bruxellensis, Brettanomyces bruxellensis, Candida californica, Candida tropicalis, Candida shehatae, Candida sylvae, Candida zemplinina, Cyberlindnera fabianii, Cyberlindnera mrakii, Cyberlindnera saturnus, Hanseniaspora uvarum, Lachancea thermotolerans, Pichia kluyveri, Pichia kudriavzevii, Saccharomyces eubayanus, Saccharomyces ludwigii, Saccharomycopsis fibuliger, Schizosaccharomyces pombe, Torulaspora delbrueckii, Wickerhamomyces anomalus, Zygoascus meyerae, Zygosaccharomyces bailii, Zygosacharomyces rouxi, Zygotorulaspora florentina*^6^

Superscripted numbers correspond to following references:

1 Roudil et al. (2019).

2 Peyer (2020), Lallemand Brewing (2021), Omega Yeast (2022), The Yeast Bay (2023), White Labs (2021), and Wyeast (2021).

3 Granchi et al. (1998), Pretorius (2000), Torija et al. (2001), Rementeria et al. (2003), Combina et al. (2005), Di Maro et al. (2007), Milanović et al. (2013), Wang and Liu (2013), Liu et al. (2016), and Bougreau et al. (2019).

4 Van Oevelen et al. (1976, 1977), Bokulich et al. (2012), Spitaels et al. (2014), Crauwels et al. (2015), Dysvik et al. (2020), Bossaert et al. (2021), and Tyakht et al. (2021).

5 Jolly et al. (2006), Viana et al. (2011), Hong and Park (2013), Benito et al. (2014), Englezos et al. (2016, 2017), and Hranilovic et al. (2020).

6 Libkind et al. (2011), Basso et al. (2016), Michel et al. (2016b), Canonico et al. (2017), Preiss et al. (2018), Bellut and Arendt (2019), Callejo et al. (2019), Canonico et al. (2019), Methner et al. (2019), Mardones et al. (2020), Urbina et al. (2020), and Larroque et al. (2021).

In contrast to whisky production, where the emphasis is on maintaining or increasing alcohol content, the wine industry seeks to lower alcohol levels due to changes in agriculture leading to grapes with excessive sugar levels. This results in high-alcohol wines with decreased flavour complexity, higher taxation, and evolving consumer preferences (Heymann et al. 2013, King et al. 2013, Saliba et al. 2013, Varela et al. 2015). As grape juice primarily consists of fructose rather than maltose, the findings of these yeast strains cannot be directly applied to whisky production.

Nevertheless, research has demonstrated that non-*Saccharomyces* yeasts significantly influence flavour production and fermentation performance, offering potential for innovation in various industries (Chatonnet et al. 1992, Romano et al. 2008, Lucy Joseph et al. 2013, Schifferdecker et al. 2014, Agnolucci et al. 2017, Berbegal et al. 2018). Given the similarity in the early production steps of Scotch Malt Whisky and beer, the knowledge gained from brewing yeast research can be more easily transferred to Scotch Malt Whisky production due to the common fermentable carbohydrate sources (Stewart et al. 2013, Bringhurst 2015, Larroque et al. 2021).

## Examples of new yeast species for Scotch Malt Whisky production

While there were 1414 accepted yeast species in 2011 (Kurtzman et al. 2011b), new yeasts are regularly found or reclassified. Currently, over 2000 yeast species and over 280 yeast genera have been identified and characterized (Boekhout et al. 2023). Unfortunately, not all of them can be discussed in this review. Table 4 provides a summary of 10 yeast species exhibiting potential as alternative Scotch Whisky distilling yeasts, as evaluated through an analysis of current literature and research (Daute 2021). Selection criteria include their ability to ferment glucose and maltose, prior use in the food industry, and a well-established research background. While less-known yeasts may also hold promise, starting with easily accessible and food-approved yeasts can simplify the initial stages of exploration.

**Table 4.** List of 10 non-conventional yeasts with the potential to be used for Scotch Whisky fermentations.

Yeast species	Frequently used synonyms	Glucose fermentation	Maltose fermentation	Origin/use	Congener production	Additional traits
*Dekkera bruxellensis* ^1^	Anamorph: *Brettanomyces bruxellensis, Brettanomyces lambicus*	Yes	Strain dependent	Beer, wine, present in biofuel production	Pharmaceutica, smoky, wet horse volatile phenols (4-vinylguaiacol, 4-ethylguaiacol), nitrogenous compounds	Production of 12% v/v ethanol, Custer effect
*Kluyveromyces lactis* ^2^	Anamorph: *Candida spherica, Saccharomyces lactis, Zygosaccharomyces lactis*	Yes	Strain dependent	Wine	Fruity, rose-like terpene production (citronellol, linalool, and geraniol)	Model organism, in cofermentations helps *Saccharomyces cerevisiae* to be more ethanol tolerant
*Lachancea thermotolerans* ^3^	*Zygosaccharomyces thermotolerans, Saccharomyces thermotolerans, Kluyveromyces thermotolerans*	Yes	Strain dependent	Beer, wine	High lactic acid, terpene, ester, glycerol	Ethanol tolerance of 5%–9% v/v, maltotriose utilization
*Wickerhamomyces anomalus* ^4^	Anamorph*: Candida pelliculosa, Pichia anomala, Hansenula anomala, Candida pelliculosa, Saccharomyces anomalus*	Yes	Strain dependent	Beer, wine, apple cider, present in malt	Fruity, sour high levels of ethyl acetate and other acetate ester, 4-vinylguaiacol, lactic acid	
*Saccharomyces bayanus* ^5^	Includes *Saccharomyces bayanus* var. *bayanus* and var. *uvarum*	Yes	Yes	Commercial wine, cider, Kveik yeast	Fruity, floral high in congeners ester (2-phenylethyl acetate, 2-methyl butanoate), and aldehydes (acetaldehyde)	Cold tolerance
*Saccharomyces paradoxus* ^6^	*Zygosaccharomyces paradoxus*	Yes	Strain dependent	Beer, wine, spontaneous aguardiente fermentation	4-Vinylguaiacol, clean flavour, like *Saccharomyces cerevisiae*	Production of 6%–12.5% v/v ethanol, deacidification in wine
*Saccharomyces pastorianus* ^7^	*Saccharomyces carlsbergensis*	Yes	Yes	Commercial beer (Lager)	Lower levels in fruity/floral and congeners compared to *Saccharomyces cerevisiae*	Well-established and researched for brewing, cold tolerance, maltotriose utilization
*Schizosaccharomyces pombe* ^8^		Yes	Yes	Whisky, beer, wine, spontaneous rum fermentation	Lower levels of congeners compared to *Saccharomyces cerevisiae*	second best studied yeast, production of 12% v/v ethanol, deacidification of wine
*Torulaspora delbrueckii* ^9^	*Saccaromyces delbrueckii, Debaryomyces delbrueckii, Zygosaccharomyces delbrueckii, Candida colliculosa, Torulaspora fermentati*	Yes	Strain dependent	Beer, wine	Low acetic acid and higher alcohols, high in esters, lactones, thiols, and terpenes	High sugar tolerance, ethanol tolerance >5% v/v
*Zygosaccharomyces rouxii* ^10^	*Saccharomyces rouxii*	Yes	Yes	Beer, spontaneous wine fermentation, soy sauce	High in higher alcohols (3-methyl-2-butanol) and aldehydes (acetaldehyde, 3-methylbutanal)	High sugar and osmotolerance

Superscripted numbers correspond to the following references:

1 Blomqvist et al. (2010), Conterno et al. (2013), and Schifferdecker et al. (2014).

2 Drawert and Barton (1978), King and Dickinson (2000), Schaffrath and Breunig (2000), Yamaoka et al. (2014), and Chen et al. (2015).

3 Domizio et al. (2016), Morata et al. (2018), and Toh et al. (2020).

4 Kurtzman (2011), Laitila et al. (2011), Ye et al. (2014), Holt et al. (2018), Osburn et al. (2018), and Padilla et al. (2018).

5 Eglinton et al. (2000), Roudil et al. (2019), Bruner and Fox (2020), and Morgan et al. (2020).

6 Pataro et al. (2000), Redžepović et al. (2002), Orlic et al. (2007), and Nikulin et al. (2020).

7 Gibson and Liti (2014), and Meier-Dörnberg et al. (2017).

8 Fahrasmane et al. (1988), Benito et al. (2016), Loira et al. (2018), Callejo et al. (2019), and Master of Malt (2021).

9 Bely et al. (2008), Canonico et al. (2016), Michel et al. (2016a), Benito (2018), Ramírez and Velázquez (2018), Toh et al. (2020), and Balmaseda et al. (2021).

10 Steels et al. (2002), Combina et al. (2005), De Francesco et al. (2015), Devanthi et al. (2018), and Escott et al. (2018).

Six of the yeast species listed (*Dekkera bruxellensis, Lachancea thermotolerans, S. bayanus, S. pastorianus, Schiz. pombe*, and *T. delbrueckii*) are commercially available, GRAS and these yeast strains have demonstrated an appropriate congener profile. Commercial strains are not available for the following yeasts, but data on several laboratory studies have been carried out: *S. paradoxus* (Pataro et al. 2000, Redžepović et al. 2002, Orlic et al. 2007, Nikulin et al. 2020), *Wickerhamomyces anomalus* (Kurtzman 2011, Laitila et al. 2011, Ye et al. 2014, Holt et al. 2018, Osburn et al. 2018, Padilla et al. 2018), and *Zygosaccharomyces rouxii* (Steels et al. 2002, Combina et al. 2005, De Francesco et al. 2015, Devanthi et al. 2018, Escott et al. 2018). For *Kluyveromyces lactis*, only a limited number of studies have been conducted in winemaking. Despite this, it is used as a model organism and possesses properties to produce higher concentrations of terpenes that could contribute to altered flavour (Drawert and Barton 1978, King and Dickinson 2000, Schaffrath and Breunig 2000, Yamaoka et al. 2014, Chen et al. 2015). A related species, *K. marxianus*, is already used in some countries to ferment cheese whey, comprising lactose, into distilled spirits and bioethanol (Grba et al. 2002, Fonseca et al. 2008).

*Saccharomyces pastorianus* and *Schiz. pombe* have been reported to produce lower levels of congeners compared to *S. cerevisiae* (Pownall et al. 2022, Benito et al. 2016, Meier-Dörnberg et al. 2017, Loira et al. 2018, Callejo et al. 2019). This characteristic could be used for lighter Scotch Whiskies, where most of the flavour originates in maturation. Alternatively, they could be used in other cereal grain-based distilled spirits where lower levels of congeners are desired such as gin or vodka. In sugarcane molasses fermentations, however, *Schiz. pombe* is known for its congener contributions to heavy flavoured dark rums.

While a wider range of non-*Saccharomyces* yeast is offered for brewing and winemaking, not all of these are able to ferment maltose. This includes *P. kluyveri, C. zemlinina, K. wickerhamii, Metschnikowia pulcherrima*, and *M. fructicola*. Since these yeasts cannot effectively convert all wort sugars, they are often sourced for brewing to produce low-alcohol beers (Johansson et al. 2021). While unsuitable for use as a pure culture in Scotch Whisky fermentations, they remain viable candidates for cofermentation, particularly in combination with *S. cerevisiae* for spirit flavour elaboration.

Several other yeast species capable of fermenting maltose have undergone laboratory studies in brewing and winemaking. Due to the scarcity of publications and nonfood safety approval, these yeasts were not included in this review. Nevertheless, it is important that other glucose and maltose fermenting yeasts such *Candida* spp., *K. dobzhanskii, L. citri, L. fermentati, M. caribbica, Scheffersomyces stipites, Schiz. japonicus, Schwanniomyces capriottii, Starmerella meliponinorum, T. franciscae, W. subpelliculosus*, or *Z. rouxii* (Kurtzman et al. 2011b) are further researched to make more yeast biodiversity available for the Scotch Whisky industry. Other yeasts, that are only able to ferment glucose but are known to be very flavourful could also be considered for cofermentation. Examples are non-*Saccharomyces* yeast used for winemaking: *C. stellata, Hanseniaspora vineae*, and *H. guilliermondii, M. pulcherrima, P. membranifaciens, P. kluyveri, W. anomalus*, or *Z. bisporus* (Ravasio et al. 2018, Postigo et al. 2022). Some of these yeasts were assessed for Scotch Whisky as part of a PhD project (Daute 2021).

**Figure 2.**
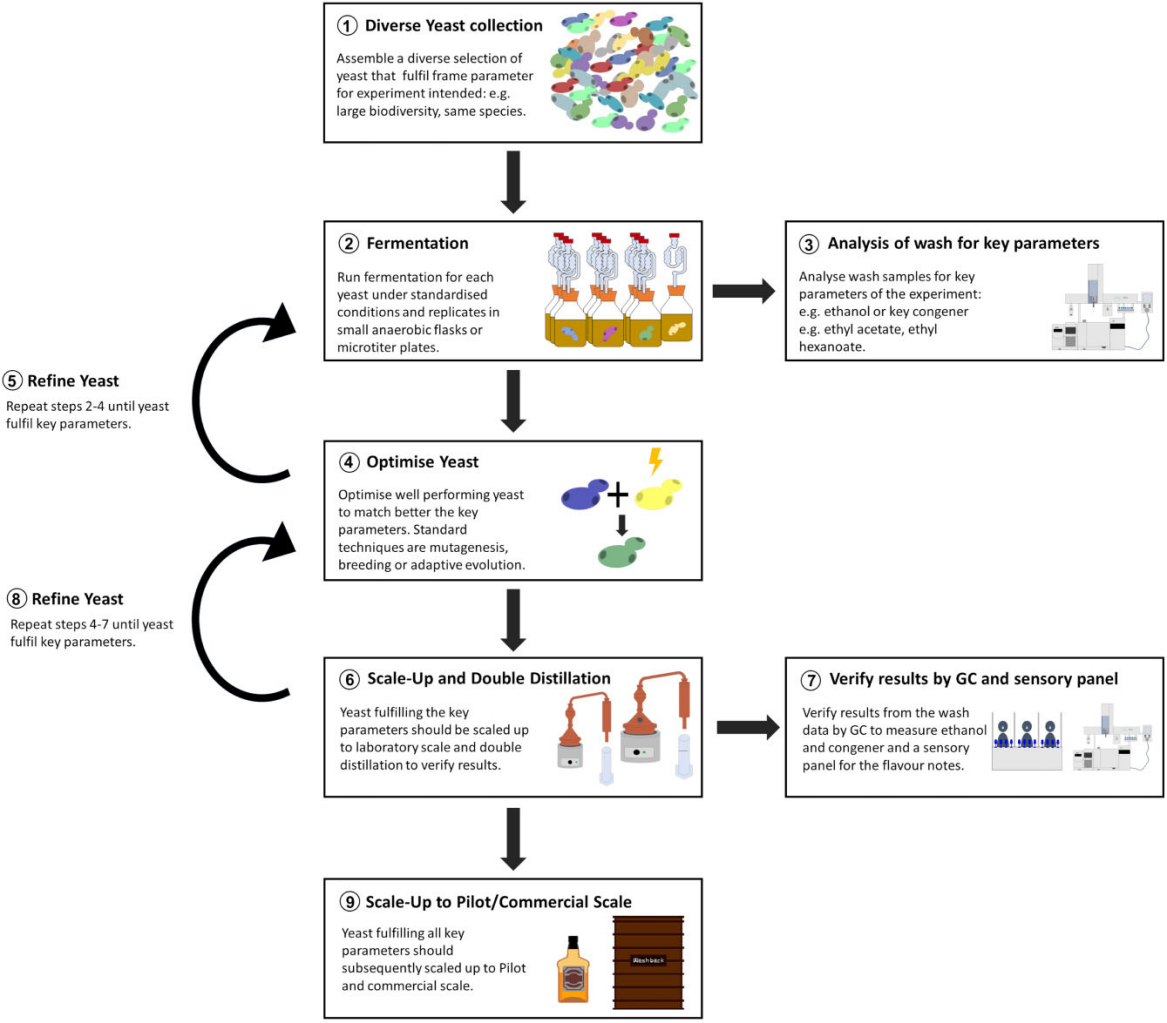
Illustrating of a strategic approach to evaluating new yeast strains.

In accordance with findings from prior research (Daute 2021), a strategic approach to evaluating new yeast strains for enhanced flavour diversification involves several steps, as depicted in Fig. 2. First, it is crucial to assemble a diverse collection of yeast from varying geographical locations, yeast species, and yeast strains, establishing a broad biodiversity. This approach aligns with observations in *S. cerevisiae*, highlighting the wide diversity in the same yeast species (Sampaio et al. 2017). Next, the yeast strains should undergo screening in small-scale fermentations, using platforms such as microtiter plates or anaerobic flasks, conducted under standardized conditions to allow comparison of fermentation results. An essential aspect of this process is the analysis of fermentation samples using GC to measure ethanol levels (indicator for fermentation performance) and congener production (indicator for flavour profile). Based on the analytical data, yeast strains with a desirable congener profile and ethanol production can be selected. Ensuring the safety of the chosen yeast strains is important before scaling further up, including assessing previous information. From this selection, a limited number of yeast strains may be selected for further optimization if necessary. The optimization phase may involve modifications to the yeast through mutagenesis, breeding, or adaptive evolution, followed by a rescreening of the strains. As the yeast strains progress, the evaluation should scale up, incorporating double distillation and sensory assessments by a panel of experts in a medium-scale fermentation setting. This iterative process repeats, until the final scale-up to large- or commercial-scale fermentations. By adhering to this systematic approach, researchers can effectively navigate the process of yeast strain selection and enhancement for the diversification of Scotch whisky flavours.

## Evaluating new yeast species and food safety qualification

Non-*Saccharomyces* yeast seem to offer a wide variety of flavour potential for the distilled spirit industry. Unfortunately, some can also be harmful by producing biogenic amines (Visciano and Schirone 2022), or some can cause opportunistic infections such as *Candida albicans* (Caetano et al. 2023). To ensure that the Scotch whisky consumption is safe, all new yeast strains, purposely added, must adhere to food safety regulation. There are two main different food safety approval systems: QPS from EFSA’s scientific panel for the European Union and GRAS from US Food and Drug Administration (FDA). Decisions are made based on the taxonomic identification, present knowledge, known safety concerns, biogenic amines, antifungal resistance, virulence, pathogenicity, and safety concerns related to the use of the yeast such as acetaldehyde production (Miguel et al. 2022). These assessments can take a long time and can be expensive. Nevertheless, this does not stop the brewing and winemaking industry from persevering with the certification of new promising yeast species for new products (Roudil et al. 2019). Recent examples of newly registered yeast strains are *P. kluyveri* from Christian Hansen (Food and Drug Administration 2020) or *M. pulcherrima* and *M. fructicola* from Lallemand (Food and Drug Administration 2021). With more and more yeast being assessed for their food safety, it can be hoped that we see further diversification in the future.

In addition to the food safety assessment, an implementation of new yeasts for Scotch Whisky also needs to adhere the Scotch Whisky Regulations (2009): Scotch whisky must ‘have the aroma and taste of Scotch Whisky’. With non-*Saccharomyces* yeast bringing new flavours into the product, it is important to ensure that the product still tastes like Scotch whisky, which limits the possible diversification.

## Conclusion

As for most distilled beverages, the considerations for Scotch Malt Whisky production revolve around ethanol yield and the overall efficiency of sugar conversion. Recent developments within the industry have witnessed distillers embracing a willingness to sacrifice ethanol yield for the creation of special-release whiskies characterized by unique and desirable flavours. Although commercial *S. cerevisiae* yeast strains continue to dominate the Scotch Whisky landscape, there exists an opportunity to draw from the trends observed in winemaking and brewing, where a diverse range of yeasts can be employed to enhance flavour profiles. Yeasts such as other *Saccharomyces* spp., *D. bruxellensis, Kluyveromyces* spp., or *Schiz. pombe* showcasing the capability to ferment primary wort sugars, demonstrate significant potential. However, using yeasts with poorer fermentation performance compared with *S. cerevisiae* distiller’s strains, can result both in reduced ethanol yields and an increase in unpleasant (e.g. sulphury) flavour notes. At the same time, other factors such as the stability of consistent fermentations, risks of unwanted contamination and ease of utilization, would need to be evaluated. In addition, it is yet unknown how any changes in new make spirit flavour profiles would pair with different oak cask types and change during maturation, although this could be predicted based on the chemical composition of the new make spirit. Looking ahead, it is predicted there will be a rise in the utilization of non-conventional yeasts and cofermentation strategies aimed at further diversifying the flavour spectrum of whiskies in the coming years. Nevertheless, these yeasts must comply with food safety regulations and the Scotch Whisky Regulations, in that the flavour profile adheres to the typical flavour of whisky.
